# Does potentially inappropriate prescribing predict an increased risk of admission to hospital and mortality? A longitudinal study of the ‘oldest old’

**DOI:** 10.1186/s12877-020-1432-4

**Published:** 2020-01-28

**Authors:** Karen Cardwell, Ngaire Kerse, Carmel M. Hughes, Ruth Teh, Simon A. Moyes, Oliver Menzies, Anna Rolleston, Joanna B. Broad, Cristín Ryan

**Affiliations:** 1 School of Pharmacy, Queen’s University Belfast, 97 Lisburn Road, Belfast, BT9 7BL Northern Ireland; 2 Department of General Practice and Primary Health Care, School of Population Health, Faculty of Medical and Health Sciences University of Auckland, Auckland, New Zealand; 3 Older People’s Health, Auckland District Health Board, Auckland, New Zealand; 4 The Centre for Health, Tauranga, New Zealand; 5 Department of Geriatric Medicine, Faculty of Medical and Health Sciences University of Auckland, Auckland, New Zealand; 6 School of Pharmacy & Pharmaceutical Science, Trinity College Dublin, The University of Dublin, College Green, Dublin, Dublin 2 Ireland

**Keywords:** Potentially inappropriate prescribing, Adults aged ≥80 years, STOPP/START criteria, Longitudinal study, Health outcomes

## Abstract

**Background:**

Potentially inappropriate prescribing (PIP) is associated with negative health outcomes, including hospitalisation and mortality. Life and Living in Advanced Age: a Cohort Study in New Zealand (LiLACS NZ) is a longitudinal study of Māori (the indigenous population of New Zealand) and non-Māori octogenarians. Health disparities between indigenous and non-indigenous populations are prevalent internationally and engagement of indigenous populations in health research is necessary to understand and address these disparities. Using LiLACS NZ data, this study reports the association of PIP with hospitalisations and mortality prospectively over 36-months follow-up.

**Methods:**

PIP, from pharmacist applied criteria, was reported as potentially inappropriate medicines (PIMs) and potential prescribing omissions (PPOs). The association between PIP and hospitalisations (all-cause, cardiovascular disease-specific and ambulatory-sensitive) and mortality was determined throughout a series of 12-month follow-ups using binary logistic (hospitalisations) and Cox (mortality) regression analysis, reported as odds ratios (ORs) and hazard ratios (HRs), respectively, and the corresponding confidence intervals (CIs).

**Results:**

Full demographic data were obtained for 267 Māori and 404 non-Māori at baseline, 178 Māori and 332 non-Māori at 12-months, and 122 Māori and 281 non-Māori at 24-months. The prevalence of any PIP (i.e. ≥1 PIM and/or PPO) was 66, 75 and 72% for Māori at baseline, 12-months and 24-months, respectively. In non-Māori, the prevalence of any PIP was 62, 71 and 73% at baseline, 12-months and 24-months, respectively. At each time-point, there were more PPOs than PIMs; at baseline Māori were exposed to a significantly greater proportion of PPOs compared to non-Māori (*p* = 0.02). In Māori: PPOs were associated with a 1.5-fold increase in hospitalisations and mortality. In non-Māori, PIMs were associated with a double risk of mortality.

**Conclusions:**

PIP was associated with an increased risk of hospitalisation and mortality in this cohort. Omissions appear more important for Māori in predicting hospitalisations, and PIMs were more important in non-Māori in predicting mortality. These results suggest understanding prescribing outcomes across and between population groups is needed and emphasises prescribing quality assessment is useful.

## Background

The prescribing of medicines is the most common medical intervention in primary care. However, research shows that the use of medicines is suboptimal [1]. As people age, they are more likely to be living with a number of chronic conditions (multimorbidity) and be prescribed a number of medications (polypharmacy) [2]. Prescribing in older people, in the context of multimorbidity, is complex as adverse effects relating to medications, e.g. drug-drug interactions and adverse drug reactions (ADRs), are more common in older age groups [3]. Consequently, potentially inappropriate prescribing (PIP) (defined as ‘over-‘, ‘under-‘ or ‘mis-prescribing’) [4] has received considerable attention in the research literature and been noted as problematic for patients and healthcare systems (e.g. hospitalisation, increased healthcare costs and mortality). However, there is inconclusive evidence relating to the long-term (> 1 year) impact (i.e. clinical, humanistic and economic) of PIP [3].

Undoubtedly, prescribing appropriateness is implicitly considered by the clinician at the point of prescribing. However, an explicit assessment of prescribing appropriateness has also evolved, through the development of prescribing tools such as Beers’ Criteria [5], Screening tool of Older Person’s Prescriptions (STOPP) and Screening Tool to Alert doctors to Right Treatment (START) version 1 [6] and version 2 [7]. The essential differences between Beers’ Criteria and STOPP/START are that the Beers’ Criteria do not include medications which are clinically indicated for a patient but are not prescribed (prescribing omissions) and include a number of medications which are absent from European formularies e.g. guanabenz and mesoridazine [7]. Using these prescribing tools, a number of studies have reported the association between PIP in older people (primarily aged 65 years and older) and health-related outcomes [3]. PIP and its effect on health trajectories may be even more significant for octogenarians due to their increased vulnerability and susceptibility to ADRs [8]. However, this has not been extensively reported in advanced age due to a paucity of clinical evidence.

Life and Living in Advanced Age: a Cohort Study in New Zealand (LiLACS NZ) is a longitudinal study of the health status of Māori (the indigenous population of New Zealand) and non-Māori octogenarians living in New Zealand [9]. Preventable health disparities between indigenous and non-indigenous populations are prevalent internationally. Health researchers have a responsibility to investigate such differences and develop initiatives to reduce this disparity and improve health outcomes for all [10]. The aim of LiLACS NZ is to explore the importance of various factors (e.g. social contact and living conditions, nutritional status, disease diagnosis, prescribed medications) in predicting health outcomes such as hospitalisations and mortality; thus enabling health services to plan and individuals to prepare for living with advanced age [9]. Using data from LiLACS NZ [9], this paper builds on previous analyses by Ryan et al. that reported the association of baseline PIP with hospitalisation and mortality at 12-months’ follow-up [11]. This study reports the association of baseline PIP with hospitalisations (categorised into all-cause, cardiovascular disease (CVD)-specific and ambulatory-sensitive hospitalisations) and mortality at 12-months’, 24-months’ and 36-months’ follow-up in a cohort of individuals aged ≥80 years.

## Methods

### Study population

LiLACS NZ, which commenced in 2010, was conceived as a bicultural study by Māori and non-Māori academics from several universities. Data from LiLACS NZ were used in this study; the complete study protocol has been published elsewhere [9]. The cohort consists of Māori (aged 80–90 years in 2010) and non-Māori (aged 85 years in 2010) recruited using multiple overlapping sampling frames to attempt a total population sample frame from a geographically defined region. Different ‘age criteria’ were applied to potential Māori and non-Māori subjects due to an observed disparity between Māori and non-Māori longevity, and because of the low numbers of Māori individuals residing in the area at the time of enrolment [9].

### Data collection

Baseline data collection involved a face-to-face standardised questionnaire (including medication data), a health assessment, an audit of general practitioner (GP) medical records and review of hospitalisation records prior to enrolment in LiLACS NZ [9]. Measures: gender, age and GP visits were ascertained by self-report at interview; prior hospitalisation from Ministry of Health records. Socioeconomic deprivation was assessed using the New Zealand Deprivation Index 2006 [12]. Medications were recorded as taken from medication containers at time of interview. Adherence was ascertained by self-report. PIP prevalence was ascertained by a pharmacist trained in the application of the criteria (KC) examining all available clinical and medication data. Functional status was assessed using the Nottingham Extended Activities of Daily Living (NEADL) scale [13]; a score ≥ 18 was classified as physically independent. Follow-up data collection involved an annual interview and health assessment. Diagnoses were ascertained using self-report, GP record review, hospitalisation discharge data and blood test analyses [14].

### Data analysis

Rongoā medicines (Māori medicines), nutritional supplements, vitamins, topical creams, those containing inactive ingredients (e.g. aqueous cream) and those taken ‘when required’ were excluded from the analysis and were not included in the assessment of prescribing appropriateness. Included medications were coded using the World Health Organisation Anatomical Therapeutic Chemical Classification system [15].

PIP prevalence was reported as potentially inappropriate medicines (PIMs) and potential prescribing omissions (PPOs) identified by STOPP and START version 1, respectively [6] as version 2 [7] had not been published at the time this study was conceived. The prevalence of PIP (i.e. ≥1 PIM, ≥1 PPO and ≥ 1 PIM and/or PPO) was reported at three time-points (baseline, 12-months and 24-months). Due to limited availability of clinical information, datasets could not be analysed for all instances of PIP noted in STOPP/START. Therefore, a sub-set of the criteria were applied to the data and some assumptions were made to facilitate the application of various criteria; see Additional file 1: Table S1 and Table S2.

### Outcomes measured

Outcomes (hospitalisations and mortality) were evaluated at 12-months’, 24-months’ and 36-months’ follow-up. Following consent, hospitalisation and mortality data were obtained annually (until death) by matching the National Health Index number (a unique identifier) with routine data on hospitalisations and mortality held by the New Zealand Ministry of Health [9]. CVD is the leading cause of mortality in the general population in New Zealand [16]. For this reason, hospitalisations were classified as all-cause, CVD-specific (identified using ICD-10 codes) and ambulatory-sensitive hospitalisations; the latter refers to hospitalisations for which effective management and treatment may prevent the admission. A standard list of diagnoses potentially susceptible to good primary care management was used to identify ambulatory-sensitive admissions [17].

### Statistical analysis

Data were analysed using Statistical Package for the Social Sciences Version 21®. Descriptive statistics provided an overview of the cohort. Attrition rates and the prevalence of PIMs and PPOs observed at each time-point were calculated. Reproducibility of results (i.e. the prevalence of PIMs and PPOs identified) by two independent researchers (trained in application of the criteria) was evaluated through calculation of Cohen’s Kappa statistic (κ) as a measure of inter-rater reliability [18]. Inter-rater reliability was interpreted as ‘poor’ if ≤0.2, ‘fair’ if 0.21–0.40, ‘moderate’ if 0.41–0.60, ‘substantial’ if 0.61–0.80 and ‘good’ if 0.81–0.99 [19].

Differences in the prevalence of PIP (i.e. PIMs and PPOs) between Māori and non-Māori were assessed using the Pearson Chi-Square (χ2) test or, for small numbers, Fisher’s Exact test (significance *p* < 0.05); differences in age and number of medicines prescribed were tested using the two-sample t-test; differences in the proportion of males and females, and socioeconomic deprivation, were tested using the χ2 test (significance *p* < 0.05).

The association between baseline PIP (as three individual binary variables i.e. ≥1 PIM, ≥1 PPO and ≥ 1 PIM and/or PPO) and hospitalisations was determined using binary logistic regression analysis, reported as odds ratios (ORs) and 95% confidence intervals (CIs), (significance *p* < 0.05). Similarly, the association between PIP and mortality was determined using Cox regression analysis, measured by hazard ratios (HRs) and 95% CIs, (significance *p* < 0.05). Regression models were adjusted for baseline age (Māori only), gender, prior hospitalisation (any hospital admission within the previous 12 months), GP visits, socioeconomic deprivation, number of medications taken and functional status as assessed by the NEADL [13].

## Results

Demographic overview and prevalence of potentially inappropriate prescribing (PIP).

Using the κ statistic, the inter-rater reliability for the identification of PIMs and PPOs (at 24-months) indicated a ‘good’ level of agreement for application of both STOPP (κ = 0.88) and START (κ = 0.80). Table 1 provides a demographic overview of the cohort and reports the prevalence of PIP and continuous PIP observed. Demographic data were obtained for 671 participants at baseline, 510 participants at 12-months and 403 participants at 24-months. Māori were significantly younger than non-Māori (*p* < 0.01). Overall, 55.7% of the cohort were female at baseline and 12-months, and 55.6% of the cohort were female at 24-months. The mean number of medicines prescribed, and the prevalence of PIP reported at each time-point was similar for Māori and non-Māori. For the combined cohort (i.e. Māori and non-Māori) the prevalence of ≥1 PIM was 26.5, 36.7 and 38.0% at baseline, 12-months and 24-months, respectively; the corresponding prevalence of ≥1 PPO was 52.6, 61.4 and 62.5%, and for ≥1 PIM and/or PPO, the prevalence was 63.5, 72.2 and 73.0% at baseline, 12-months and 24-months, respectively; see Table 1 for Māori and non-Māori data presented separately.

**Table 1:** Demographic overview and exposure to potentially inappropriate prescribing for all individuals enrolled in LiLACS NZ at each time point

	Baseline (*n* = 671)			12-months (*n* = 510)			24-months (*n* = 403)		
	Māori (*n* = 267)	Non-Māori (*n* = 404)	*P* value	Māori (*n* = 178)	Non-Māori (*n* = 332)	P value	Māori (*n* = 122)	Non-Māori (*n* = 281)	P value
Age (years, mean ± SD)	82.27 ± 2.64	84.56 ± 0.53	< 0.01*	83.16 ± 2.60	85.52 ± 0.51	< 0.01*	84.22 ± 2.58	86.54 ± 0.51	< 0.01*
Female n (%)	160 (59.9)	214 (53.0)	0.50†	107 (60.1)	177 (53.3)	0.14†	78 (63.9)	146 (52.0)	0.03†
Number of all medicines prescribed (mean ± SD)	4.63 ± 3.24	4.92 ± 3.18	0.25*	5.38 ± 3.57	5.29 ± 3.33	0.78*	5.69 ± 3.53	5.56 ± 3.34	0.68*
Socioeconomic deprivation (NZDep) n (%) 0–4 5–7 8–10	37 (13.9) 65 (24.3) 165 (61.8)	101 (25.0) 171 (42.3) 132 (32.7)	< 0.01‡	27 (15.2) 46 (25.8) 105 (59.0)	83 (25.0) 143 (43.1) 106 (31.3)	< 0.01‡	16 (13.1) 35 (28.7) 71 (58.2)	71 (25.3) 124 (44.1) 86 (30.6)	< 0.01‡
Individuals with ≥1 PIM n (%)	65 (24.3)	113 (28.0)	0.17†	62 (34.8)	125 (37.7)	0.53†	43 (35.3)	110 (39.2)	0.46†
Individuals with ≥1 PPO n (%)	155 (58.1)	198 (49.0)	0.01†	119 (66.9)	194 (58.4)	0.06†	80 (65.6)	172 (61.2)	0.41†
Individuals with ≥1 PIM and/or PPO n (%)	175 (65.5)	251 (62.1)	0.20†	134 (75.3)	234 (70.5)	0.25†	88 (72.1)	206 (73.3)	0.81†

Key: NZDep, New Zealand Deprivation Index score (higher NZDep score is more deprived); *Two-samples t-test (significance *p* < 0.05); †Chi-squared (χ2) test (significance *p* < 0.05); ‡Mann Whitney U test (significance *p* < 0.05); *P* values measure differences between Māori and non-Māori

Of the medical conditions recorded, the incidence of most was similar in Māori and non-Māori. However, chronic heart failure (CHF), type 2 diabetes, asthma/chronic lung disease and rheumatoid arthritis were markedly more common in Māori individuals at baseline, 12-months and 24-months, compared to non-Māori; the incidence of osteoarthritis was substantially greater in non-Māori individuals at baseline, 12-months and 24-months, compared to Māori. See Table 2 for an overview of the medical condition diagnoses for all individuals enrolled in LiLACS NZ at each time-point.

**Table 2:** Medical condition diagnoses for all individuals enrolled in LiLACS NZ at each time-point

	Baseline (*n* = 671)		12-months (*n* = 510)		24-months (*n* = 403)	
Medical Condition	Māori *n* = 267	Non-Māori *n* = 404	Māori *n* = 178	Non-Māori *n* = 332	Māori *n* = 122	Non-Māori *n* = 281
Hypertension	232	350	166	295	114	250
Chronic heart failure	81	7	52	61	36	58
Atrial fibrillation	73	118	37	66	28	52
Rheumatoid arthritis	71	49	51	39	38	38
Osteoarthritis	68	163	44	143	33	125
Depression	66	38	17	33	10	15
Type 2 diabetes	58	51	40	40	23	34
Cerebrovascular disease	56	105	36	82	25	66
Asthma/Chronic lung disease	50	49	32	30	20	24
Peripheral vascular disease	37	54	25	42	17	30
Osteoporosis	32	68	36	73	24	62
Dementia	13	13	21	25	16	19
Thyroid disease	8	23	2	10	2	11
Renal impairment	4	4	3	3	2	3
Parkinson’s disease	2	9	1	3	0	2

A description of the most common PIMs and PPOs observed at baseline has been reported previously [11]. At 12-months and 24-months, the most common PIMs overall were ‘a proton pump inhibitor (PPI) at full therapeutic dose for more than 8 weeks’, and ‘long-term opiates in those with recurrent falls’. Two prescribing scenarios were more common in Māori participants: ‘a thiazide in those with a history of gout’ and ‘diltiazem or verapamil in those with New York Heart Association class III or IV heart failure’; see Additional file 1: Table S3.

In both Māori and non-Māori, the most common PPO at 12-months and 24-months was the omission of ‘a calcium and vitamin D supplement in patients with known osteoporosis’. In Māori participants, omission of ‘an angiotensin-converting-enzyme inhibitor (ACE) inhibitor in those with congestive heart failure (CHF)’ was more common than in non-Māori participants. Conversely, the omission of ‘statin therapy in those with a documented history of coronary, cerebral or peripheral vascular disease’ was more common in non-Māori participants compared to Māori participants; see Additional file 1: Table S4.

See Additional file 1: Table S5 and Table S6 for an overview of the prevalence of PIMs and PPOs within each physiological system, identified by STOPP and START, respectively for all individuals enrolled in LiLACS NZ.

### Potentially inappropriate prescribing (PIP) and health-related outcomes

#### Māori cohort

Table 3 provides an overview of the association between exposure to PIP and health-related outcomes in the Māori cohort. Exposure to ≥1 PPO was associated with an increased risk of CVD-specific hospitalisations at 12-months’ follow-up, ambulatory-sensitive hospitalisations at 12-months’ and 24-months’ follow-up and mortality at 24-months’ and 36-months’ follow-up. Exposure to ≥1 PIM and/or PPO was associated with an increased risk of CVD-specific and ambulatory-sensitive hospitalisations at 12-months’ follow up, all-cause hospitalisation at 24-months’ follow-up and mortality at 36-months’ follow-up; see Table 3.

**Table 3:** The association between potentially inappropriate prescribing at baseline and outcomes at 12, 24 and 36-months’ follow-ups for all Māori individuals

	Exposure to PIP at baseline	12-months’ follow-up	24-months’ follow-up	36-months’ follow-up
Increased risk of all-cause hospitalisation OR (95% CI)† *p* value*	≥1 PIM	0.95 (0.45, 2.02) 0.90	1.42 (0.69, 2.95) 0.34	1.10 (0.41, 2.96) 0.85
	≥1 PPO	1.66 (0.83, 3.31) 0.15	1.59 (0.89, 2.83) 0.12	2.16 (0.99, 4.72) 0.06
	≥1 PIM and/or PPO	1.66 (0.79, 3.46) 0.18	1.88 (1.04, 3.40) 0.04	1.69 (0.79, 3.62) 0.18
Increased risk of CVD-specific hospitalisation OR (95% CI)† *p* value*	≥1 PIM	1.26 (0.60, 2.64) 0.54	1.50 (0.75, 3.00) 0.25	1.48 (0.65, 3.36) 0.35
	≥1 PPO	2.86 (1.37, 5.95) 0.01	1.50 (0.85, 2.66) 0.16	1.45 (0.77, 2.72) 0.25
	≥1 PIM and/or PPO	2.98 (1.34, 6.64) 0.01	1.52 (0.85, 2.75) 0.16	1.18 (0.62, 2.24) 0.61
Increased risk of ambulatory sensitive hospitalisation OR (95% CI)† *p* value*	≥1 PIM	1.30 (0.65, 2.58) 0.46	1.14 (0.56, 2.34) 0.72	0.92 (0.39, 2.14) 0.84
	≥1 PPO	2.31 (1.22, 4.37) 0.01	1.97 (1.11, 3.51) 0.02	1.74 (0.89, 3.38) 0.10
	≥1 PIM and/or PPO	2.14 (1.09, 4.19) 0.03	1.77 (0.99, 3.19) 0.06	1.37 (0.71, 2.67) 0.35
Mortality HR (95% CI)‡ *p* value*	≥1 PIM	1.08 (0.39, 3.03) 0.88	1.21 (0.59, 2.45) 0.61	1.48 (0.90, 2.44) 0.12
	≥1 PPO	3.44 (0.73, 16.33) 0.12	2.53 (1.08, 5.94) 0.03	2.07 (1.23, 3.48) 0.01
	≥1 PIM and/or PPO	1.70 (0.36, 7.99) 0.50	2.46 (0.92, 6.55) 0.07	2.11 (1.18, 3.79) 0.01

Key: CI, Confidence Interval; CVD, Cardiovascular disease; HR, hazard ratio; PIM, potentially inappropriate medicine; PIP, potentially inappropriate prescribing; PPO, potential prescribing omission; OR, odds ratio; †Binary logistic regression (significance *p* < 0.05); ‡Cox regression (significance *p* < 0.05); *Adjusted for baseline age, gender, GP visits, prior hospitalisation (within previous 12 months), socioeconomic deprivation, number of medicines prescribed, functional status (Nottingham Extended Activities of Daily Living score)

#### Non-Māori cohort

Table 4 provides an overview of the association between exposure to PIP and health-related outcomes in the non-Māori cohort. Exposure to ≥1 PIM (and exposure to ≥1 PIM and/or PPO) was associated with an increased risk of mortality at 36-months’ follow-up; see Table 4.

**Table 4:** The association between potentially inappropriate prescribing at baseline and outcomes at 12, 24 and 36-months’ follow-ups for all non-Māori individuals

	Exposure to PIP at baseline	12-months’ follow-up	24-months’ follow-up	36-months’ follow-up
Increased risk of all-cause hospitalisation OR (95% CI)† *p* value*	≥1 PIM	1.17 (0.70, 1.96) 0.55	0.96 (0.59, 1.57) 0.87	0.71 (0.40, 1.45) 0.23
	≥1 PPO	1.11 (0.69, 1.77) 0.68	1.33 (0.86, 2.03) 0.20	0.90 (0.56, 1.47) 0.68
	≥1 PIM and/or PPO	1.10 (0.67, 1.82) 0.71	1.09 (0.70, 1.70) 0.71	0.81 (0.49, 1.33) 0.40
Increased risk of CVD-specific hospitalisation OR (95% CI)† *p* value*	≥1 PIM	1.36 (0.74, 2.50) 0.32	1.19 (0.73, 1.94) 0.48	1.00 (0.62, 1.63) 0.99
	≥1 PPO	1.08 (0.60, 1.94) 0.80	1.45 (0.93, 2.24) 0.10	1.14 (0.75, 1.74) 0.55
	≥1 PIM and/or PPO	1.52 (0.78, 2.96) 0.22	1.60 (1.00, 2.57) 0.05	1.15 (0.74, 1.79) 0.54
Increased risk of ambulatory sensitive hospitalisation OR (95% CI)† *p* value*	≥1 PIM	1.17 (0.69, 1.98) 0.55	1.44 (0.89, 2.34) 0.14	1.21 (0.72, 2.03) 0.47
	≥1 PPO	1.12 (0.69, 1.81) 0.65	1.44 (0.94, 2.20) 0.09	1.04 (0.67, 1.61) 0.87
	≥1 PIM and/or PPO	1.07 (0.64, 1.79) 0.79	1.42 (0.91, 2.21) 0.12	1.04 (0.66, 1.64) 0.86
Mortality HR (95% CI)‡ *p* value*	≥1 PIM	1.32 (0.49, 3.58) 0.58	1.41 (0.76, 2.62) 0.27	1.53 (1.02, 2.32) 0.04
	≥1 PPO	2.21 (0.69, 7.03) 0.18	1.29 (0.71, 2.35) 0.40	1.40 (0.93, 2.10) 0.10
	≥1 PIM and/or PPO	6.19 (0.79, 48.61) 0.08	1.68 (0.83, 3.39) 0.15	1.61 (1.01, 2.57) < 0.05

Key: CI, Confidence Interval; CVD, Cardiovascular disease; HR, hazard ratio; PIM, potentially inappropriate medicine; PIP, potentially inappropriate prescribing; PPO, potential prescribing omission; OR, odds ratio; †Binary logistic regression (significance *p* < 0.05); ‡Cox regression (significance *p* < 0.05); *Adjusted for baseline age, gender, GP visits, prior hospitalisation (within previous 12 months), socioeconomic deprivation, number of medicines prescribed, functional status (Nottingham Extended Activities of Daily Living score)

## Discussion

Using data from LiLACS NZ, this study reported the prevalence of PIP (defined by STOPP/START) at three time-points (baseline, 12-months and 24-months) and explored the association of baseline PIP with outcomes (all-cause, CVD-specific and ambulatory-sensitive hospitalisations and mortality) at 12-month intervals (12-months’, 24-months’ and 36-months’ follow-up). PIP was highly prevalent, and PPOs were more common than PIMs. In Māori, PPOs were associated with at least one outcome (increased risk of hospitalisation or mortality) at each time-point; in non-Māori, PIMs were associated with an increased risk of mortality at 36-months’ follow-up only. This is one of a few studies to find a prospective association between PIP (defined by STOPP/START) and health outcomes. In the context of population ageing, such data are increasingly relevant to forward planning of health services. This study adds evidence from the southern hemisphere to that from Europe, the USA and Taiwan [3] for the utility of STOPP/START in identifying PIP associated prospectively with adverse outcomes. We add that the associations between inappropriate prescribing and increased risk of hospitalisation and mortality persist into advanced age (≥80 years) and that PPOs may be as, and perhaps more, important than PIMs.

Considering that there were few differences between Māori and non-Māori in relation to the levels of polypharmacy observed, it is intriguing that the association between PIP and the described health-related outcomes differ. This could potentially be explained by the differing patterns of multimorbidity observed in the two ethnic groups [20]. The association between prescribing omissions and CVD hospitalisations is particularly relevant. The prevalence of CHF and Diabetes mellitus were greater amongst Māori [21] and the increased prevalence of PPOs potentially suggests under-treatment with ACE inhibitor in CHF of Māori in advanced age. Disparities in access and outcomes related to CVD for Māori are well known [22–26] and these appear to persist into advanced age. This finding supports the need to individualise approaches to treatment for older people from diverse backgrounds with a call for more specific research in different ethnic groups. Moreover, the issue of institutional racism in treatment also needs to be raised where outcomes differ through systematic differences in treatment patterns between ethnic groups [27, 28].

In accordance with previous studies of PIP [3], the most frequently encountered PIMs were the prolonged use of high-dose PPIs, as well as opiates in those at risk of falling. Although PPIs have a favourable risk-benefit ratio, their use should be reviewed regularly as there are concerns surrounding an increased risk of infections and reduced absorption of nutrients with long term PPI use, in particular vitamin B12 and calcium [29]. In this study, the prescription of benzodiazepines, tricyclic antidepressants, anticholinergics and opiates increased with time. Such drugs are a significant problem in older people due to the possibility of dependence and association with side effects such as falls, confusion, dizziness and constipation [30]; this study exemplifies the challenges of safely managing multimorbidity in the elderly.

The omission of a calcium and vitamin D supplement in this study also increased substantially with time, which may reflect the local uncertainty surrounding its safety in the presence of CVD. Current evidence regarding vitamin D and, particularly, calcium supplementation is inconclusive; New Zealand has been the source of strong debate [31, 32] and local prescribers may have been more influenced than international trends suggest. The suggestion that medicines (particularly those associated with CVD) cause more harm than benefit in older people is a clinical conundrum when prescribing for this population group. The use of antihypertensives (in those aged ≥80 years) [33] and statins (in those aged 40–80 years and 70–82 years) [34] has been shown to be beneficial in secondary prevention of CVD in older people. However, uncertainty remains about the benefits of statin use for primary prevention [35]. This is particularly important in this older, more vulnerable population group as they are more susceptible to the adverse effects of drugs [8]. Potentially, this study suggests that conservative prescribing for CVD risk may not be in the best interests of those in advanced age, given the omission of CVD related medicines observed in this cohort. However, clinical trials of conservative versus comprehensive prescribing for multimorbidity are needed before causality can be claimed.

In this cohort, prescribing omissions were more common in Māori than non-Māori. Reasons for this disparity have not been investigated in this study but are complex and associated with system-based issues such as access [22]. A large body of evidence has also identified institutional racism as a cause of health inequalities for Māori in New Zealand. Thus, there is a need for on-going strategies to ensure Māori are not marginalised in health [27, 28]. The association between PIP and mortality observed in this cohort is not consistent over time, nevertheless, it suggests the need for trials to test the efficacy of prescribing strategies.

Recruitment to LiLACS NZ was favourable; of those contacted, 64% agreed to participate (*n* = 937) at baseline. The inability to engage ethnic minority groups in research is common; this may have been overcome by the support of a Māori oversight group, ‘Rōpū Kaitiaki o tikanga Māori’ [36]. Comparisons between LiLACS NZ and other population-based samples suggests that LiLACS NZ data largely reflects the older population of New Zealand. However, it should be noted that non-Māori living in residential care may be underrepresented [37]. Moreover, prescribing practices have been shown to differ across New Zealand and globally. Therefore, the generalisability of the results may be limited. Despite this, these results serve as an important comparator for other longitudinal studies of PIP. LiLACS NZ data collection was comprehensive and data collectors were trained by researchers who were experienced in engaging with older people. However, data collection incurred a high participation burden and as a result, 28% of those recruited opted to complete a shorter interview that did not include medication use. Length of follow-up is a major strength of this study since previous studies of PIP have had short follow-up periods [38]. The attrition rate between the two time-points was 21% and is an inevitable limitation of ageing research, i.e. attrition rates are higher than in studies of younger populations. Overall small numbers will limit this analysis and the possibility of a missing a significant association (Type II error) is high.

The LiLACS NZ dataset was information-rich and included medication data as well as clinical information. Medication use was ascertained from medication containers provided by study participants which provides a more reliable indication of medication use compared to electronic dispensing records. However, adherence was ascertained by self-report which is subject to reporting bias. Moreover, Rongoā medicines (Māori medicines) were omitted from the analysis, thus the association between these medicines and outcomes was not assessed. The use of clinical information, in addition to medication data, helped prevent the overestimation of PIP as participants’ co-morbidities and clinical picture were taken into consideration. The diagnosis of chronic conditions was verified using GP records, but since this was completed at baseline only, data collectors relied on the ability of participants to report any clinical diagnoses made thereafter. Consequently, the true incidence of clinical conditions may have been underestimated, and thus the prevalence of PIP. Increasingly, patient involvement in the prescribing process is being advocated. However, due to the design of this study it was not possible to account for patient preferences when identifying issues of PIP. Other limitations include the inability to apply all STOPP/START criteria and the use of proxies (assumptions) to facilitate the application of certain criteria; these limitations are common to most studies of PIP. The estimation of ambulatory-sensitive hospitalisations may be inaccurate in this age group as criteria were developed for use in those aged up to 75 years [17]. Finally, although this study reports a significant association between exposure to PIP and an increased risk of admission to hospital, this does not infer causality due to the potential influence of residual confounding [39], e.g. presence of co-morbidities.

## Conclusions

PIP was highly prevalent in this cohort of individuals, aged ≥80 years living in New Zealand, and associated with an increased risk of hospitalisations and mortality. Omissions in medication were more common than inappropriate medication use, particularly for Māori. Given the predicted change in global demographics, these results are important in full understanding of the relationship between PIP and poor health outcomes.

## Supplementary information

**Additional file 1: Table S1.** Assumptions used in the application of STOPP criteria to LiLACS NZ data. **Table S2.** Assumptions used in the application of START to LiLACS NZ data. **Table S3.** Potentially inappropriate medicines (PIMs) identified by STOPP, for all individuals enrolled in LiLACS NZ at 12-months and 24-months. **Table S4.** Potential prescribing omissions (PPOs) identified by START, for all individuals enrolled in LiLACS NZ at 12-months and 24-months. **Table S5.** The prevalence of Potentially inappropriate medicines (PIMs) within each physiological system, identified by STOPP, for all individuals enrolled in LiLACS NZ at 12-months and 24-months. **Table S6.** The prevalence of Potential prescribing omissions (PPOs) within each physiological system, identified by START, for all individuals enrolled in LiLACS NZ at 12-months and 24-months.

## Data Availability

The datasets generated and/or analysed during the current study are not publicly available as data collection and analysis is on-going but are available from the corresponding author on reasonable request.

## Abbreviations

ACE: Angiotensin converting enzyme
ADR: Adverse drug reaction
CHF: Chronic heart failure
CI: Confidence interval
CVD: Cardiovascular disease
HR: Hazard ratio
LiLACS NZ: Life and Living in Advanced Age: a Cohort Study in New Zealand
NEADL: Nottingham Extended Activities of Daily Living
OR: Odds ratio
PIM: Potentially inappropriate medicine
PIP: Potentially inappropriate prescribing
PPI: Proton pump inhibitor
PPO: Potential prescribing omission
START: Screening Tool to Alert doctors to Right Treatment
STOPP: Screening Tool of Older Persons’ Prescriptions
