# Supplementary material for: Does potentially inappropriate prescribing predict an increased risk of admission to hospital and mortality? A longitudinal study of the ‘oldest old’
Source: BMC Geriatr. 2020 Jan 28;20:28. doi: 10.1186/s12877-020-1432-4 (PMC6986145; doi:10.1186/s12877-020-1432-4)
Supplement: Supplementary file 1 — Additional file 1: Table S1. Assumptions used in the application of STOPP criteria to LiLACS NZ data. Table S2. Assumptions used in the application of START to LiLACS NZ data. Table S3. Potentially inappropriate medicines (PIMs) identified by STOPP, for all individuals enrolled in LiLACS NZ at 12-months and 24-months. Table S4. Potential prescribing omissions (PPOs) identified by START, for all individuals enrolled in LiLACS NZ at 12-months and 24-months. Table S5. The prevalence of Potentially inappropriate medicines (PIMs) within each physiological system, identified by STOPP, for all individuals enrolled in LiLACS NZ at 12-months and 24-months. Table S6. The prevalence of Potential prescribing omissions (PPOs) within each physiological system, identified by START, for all individuals enrolled in LiLACS NZ at 12-months and 24-months. [file 12877_2020_1432_MOESM1_ESM.docx]

**Table S1 Assumptions used in the application of STOPP criteria to LiLACS NZ data**

| STOPP criteria | Assumptions |
| --- | --- |
| Cardiovascular System | |
| Digoxin at a long-term dose >125 μg/day with impaired renal function | NA |
| Loop diuretic for dependent ankle oedema only | NA |
| Loop diuretic as first-line monotherapy for hypertension | NA |
| Thiazide diuretic with a history of gout | Prescribing of allopurinol/colchicine indicates gout |
| Non-cardioselective β-blocker with COPD | NA |
| β-blocker in combination with verapamil | NA |
| Use of diltiazem or verapamil with NYHA class III or IV heart failure | Prescribing of digoxin indicates class III or IV heart failure |
| CCBs with chronic constipation | Prescribing of laxatives at previous time-point indicates chronic constipation |
| Aspirin and warfarin without H_2_-receptor antagonist | NA |
| Dipyridamole as monotherapy for cardiovascular secondary prevention | NA |
| Aspirin at dose >150 mg/day | NA |
| Aspirin with no history of coronary, cerebral or peripheral vascular symptoms or occlusive event | NA |
| Aspirin to treat dizziness not clearly attributable to cerebrovascular disease | NA |
| Central Nervous System | |
| TCAs with dementia | NA |
| TCAs with glaucoma | Prescribing of topical glaucoma treatments indicates glaucoma |
| TCAs with cardiac conductive abnormalities | Prescribing of medications indicated for arrhythmias indicates conductive abnormalities |
| TCAs with constipation | NA |
| TCAs with an opiate or CCB | NA |
| Long-term (i.e. >1 month), long-acting benzodiazepines or benzodiazepines with long-acting metabolites | Prescribing of long-acting benzodiazepines at previous time-point indicates long-term treatment |
| Long-term neuroleptics (>1 month) in those with Parkinson’s Disease | Prescribing of neuroleptics at previous time-point indicates long-term treatment |
| Phenothiazines in patients with epilepsy | NA |
| Anticholinergic medications to treat extrapyramidal side-effects of neuroleptic medications | NA |
| SSRls with a history of clinically significant hyponatremia | Hyponatremia recorded at previous time-point indicates clinically significant hyponatremia |
| Prolonged use (>1 week) of first-generation antihistamines | Prescribing of first-generation antihistamine at previous time-point indicates prolonged use |
| Prochlorperazine (Stemetil) or metoclopramide with Parkinson’s Disease | NA |
| Gastrointestinal System | |
| PPI for peptic ulcer disease at full therapeutic dosage for >8 weeks | Prescribing of PPI at previous time-point indicates treatment >8 weeks |
| Anticholinergic antispasmodic drugs with chronic constipation | Prescribing of laxatives at previous time-point indicates chronic constipation |
| Respiratory System | |
| Theophylline as monotherapy for COPD | NA |
| Systemic corticosteroids instead of inhaled corticosteroids for maintenance therapy in moderate-to-severe COPD | COPD diagnosis recorded indicates moderate-to-severe COPD |
| Nebulized ipratropium with glaucoma | Prescribing of topical glaucoma treatments indicates glaucoma |
| Musculoskeletal System | |
| NSAID with moderate-to-severe hypertension | BP measurements ≥140/90mmHg indicates moderate-to-severe hypertension |
| NSAID with heart failure | NA |
| Long-term NSAID (>3 months) for symptom relief of mild osteoarthritis | Prescribing of NSAID and recorded osteoarthritis at previous time-point indicates long-term symptom relief |
| Warfarin and NSAID together | NA |
| NSAID with chronic renal failure | NA |
| Long-term corticosteroids (>3 months) as monotherapy for rheumatoid arthritis or osteoarthritis | Prescribing of systemic corticosteroid as monotherapy and recorded rheumatoid arthritis or osteoarthritis at previous time-point indicates therapy >3 months |
| Long-term NSAID or colchicine for chronic treatment of gout where there is no contraindication to allopurinol | Prescribing of allopurinol/colchicine indicates gout |
| Urogenital System | |
| Bladder anticholinergic medications with dementia | NA |
| Anticholinergic medications with chronic glaucoma | Prescribing of topical glaucoma treatments indicates glaucoma |
| Anticholinergic medications with chronic constipation | Prescribing of laxatives at previous time-point indicates chronic constipation |
| Anticholinergic medications with chronic prostatism | Prostatism recorded at previous time-point indicates chronic prostatism |
| Endocrine System | |
| Glibenclamide or chlorpropamide with type 2 diabetes mellitus | Prescribing of antidiabetic medications indicates diabetes mellitus diagnosis |
| β-blockers in those with diabetes mellitus and frequent hypoglycemic episodes i.e. ≥1 episode per month | Hypoglycaemia recorded at previous time-point indicates frequent episodes of hypoglycaemia |
| Estrogens with a history of breast cancer or venous thromboembolism | NA |
| Fall-inducing Drugs | |
| Benzodiazepines | NA |
| Neuroleptic medications | NA |
| First-generation antihistamines | NA |
| Vasodilator drugs with persistent postural hypotension, i.e. recurrent >20mmHg drop in systolic blood pressure | Recorded history of falls |
| Long-term opiates in those with recurrent falls | Recorded history of falls and prescribing of opiates at previous time-point |
| Opiates | |
| Use of long-term powerful opiates first-line for mild-to-moderate pain | NA |
| Regular opiates for >2 weeks in those with chronic constipation without concurrent use of laxatives | Prescribing of laxatives at previous time-point indicates chronic constipation |
| Duplicate Prescribing | |
| Any duplicate drug class prescription | NA |

Key: BP, blood pressure; CCB, Calcium channel blocker; CNS, central nervous system; COPD, chronic obstructive pulmonary disease; CVS, cardiovascular system; ES, endocrine system; GIS, gastrointestinal system; MS, musculoskeletal system; NA, Not applicable; NSAID, Non-steroidal anti-inflammatory drug; NYHA, New York Heart Association; PPI, proton pump inhibitor; RS, respiratory system; SSRl, Selective serotonin re-uptake inhibitor; TCA, Tricyclic antidepressant; US, urogenital system.

**Table S2 Assumptions used in the application of START to LiLACS NZ data**

| START criteria | Assumptions |
| --- | --- |
| Cardiovascular System | |
| Warfarin or aspirin in the presence of chronic atrial fibrillation | NA |
| Aspirin or clopidogrel with a documented history of atherosclerotic coronary, cerebral or peripheral vascular disease in patients with sinus rhythm | NA |
| Antihypertensive therapy where systolic blood pressure consistently >160 mmHg | NA |
| Statin therapy with a documented history of coronary, cerebral or peripheral vascular disease, where the patient's functional status remains independent for activities of daily living and life expectancy is greater than 5 years | NEADL score ≥18 indicates independent physical functioning |
| ACE inhibitor with chronic heart failure | Recorded chronic heart failure at previous time-point indicates chronic heart failure |
| ACE inhibitor following acute MI | No record of MI at previous time-point indicates acute MI |
| β-blocker with chronic stable angina | Recorded angina indicates stable |
| Central Nervous System | |
| Antidepressant drug in moderate-severe depressive symptoms ≥ 3 months | GDS score >5 indicates moderate-severe depressive symptoms |
| Gastrointestinal System | |
| Fibre supplement for chronic, symptomatic diverticular disease with constipation | Recorded diverticular disease at previous time-point indicates chronic diverticular disease |
| Musculoskeletal System | |
| Bisphosphonates in patients taking maintenance corticosteroid therapy | Prescribing of systemic corticosteroid therapy at previous time-point indicates maintenance therapy |
| Calcium and vitamin D supplement in patients with known osteoporosis | NA |
| Endocrine System | |
| Metformin with type 2 DM ± metabolic syndrome (in absence of renal impairment) | NA |
| ACE inhibitor or angiotensin receptor blocker in diabetes with nephropathy, i.e. overt urinalysis proteinuria or microalbuminuria (>30 mg/24 hours) ±serum biochemical renal impairment | NA |
| Antiplatelet therapy in DM with coexisting major cardiovascular risk factors | Major risk factors defined as smoking, BP≥140/90mmHg, CVD diagnosis |
| Statin therapy in DM if coexisting major cardiovascular risk factors present | Major risk factors defined as smoking, BP≥140/90mmHg, CVD diagnosis |

Key: ACE, angiotensin converting enzyme; BP, blodd pressure; CNS, central nervous system; COPD, chronic obstructive pulmonary disease; CVD, cardiovascular disease; CVS, cardiovascular system; DM, diabetes mellitus; ES, endocrine system; GIS, gastrointestinal system; GDS, Geriatric depression scale; MI, myocardial infarction; MS, musculoskeletal system; NA, not applicable; Nottingham Extended Activities of Daily Living (NEADL).

**Table S3 Potentially inappropriate medicines (PIMs) identified by STOPP, for all individuals enrolled in LiLACS NZ at 12-months and 24-months**

| STOPP Criteria | 12-months  (n=510) | | 24-months  (n=403) | |
| --- | --- | --- | --- | --- |
|  | Māori  (n=178) | Non-Māori  (n=332) | Māori  (n=122) | Non-Māori  (n=281) |
| Cardiovascular System | | | | |
| Digoxin at a long-term dose >125 μg/day with impaired renal function | 0 (0.0) | 0 (0.0) | 0 (0.0) | 0 (0.0) |
| Loop diuretic for dependent ankle oedema only | 0 (0.0) | 1 (0.3) | 0 (0.0) | 1 (0.4) |
| Loop diuretic first line monotherapy for hypertension | 1 (0.6) | 9 (2.7) | 0 (0.0) | 6 (2.1) |
| Thiazide diuretic with a history of gout | 7 (3.9) | 0 (0.0) | 4 (3.3) | 1 (0.4) |
| Non-cardioselective β-Blocker with COPD | 1 (06) | 2 (0.60 | 2 (1.6) | 1 (0.4) |
| β-blocker in combination with verapamil | 0 (0.0) | 0 (0.0) | 1 (0.8) | 0 (0.0) |
| Use of diltiazem or verapamil with NYHA class III or IV heart failure | 10 (5.6) | 2 (0.6) | 3 (2.5) | 1 (0.4) |
| CCB with chronic constipation | 13 (7.3) | 18 (5.4) | 11 (9.0) | 15 (5.3) |
| Aspirin and warfarin without H_2_-receptor antagonist | 1 (0.6) | 0 (0.0) | 0 (0.0) | 2 (0.7) |
| Dipyridamole as monotherapy for cardiovascular secondary prevention | 0 (0.0) | 1 (0.3) | 0 (0.0) | 0 (0.0) |
| Aspirin at a dose >150mg/day | 1 (0.6) | 3 (0.9) | 1 (0.8) | 1 (0.4) |
| Aspirin with no hx of coronary/cerebral/peripheral vascular symptoms or occlusive event | 0 (0.0) | 0 (0.0) | 0 (0.0) | 0 (0.0) |
| Central Nervous System | | | | |
| TCAs with dementia | 1 (0.6) | 1 (0.3) | 1 (0.8) | 3 (1.1) |
| TCAs with glaucoma | 0 (0.0) | 3 (0.9) | 1 (0.8) | 4 (1.4) |
| TCAs with cardiac conductive abnormalities | 2 (1.1) | 5 (1.5) | 2 (1.6) | 7 (2.5) |
| TCAs with constipation | 1 (0.6) | 6 (1.8) | 1 (0.8) | 5 (1.8) |
| TCAs with opiate or CCB | 4 (2.2) | 9 (2.7) | 5 (4.1) | 13 (4.6) |
| Long-term (i.e. >1 month), long-acting benzodiazepines | 2 (1.1) | 5 (1.5) | 2 (1.6) | 4 (1.4) |
| Long-term neuroleptics (>1 month) in those with Parkinson’s Disease | 0 (0.0) | 0 (0.0) | 0 (0.0) | 0 (0.0) |
| Anticholinergic medications to treat side effects of neuroleptic medications | 0 (0.0) | 2 (0.60 | 0 (0.0) | 0 (0.0) |
| SSRI with a history of clinically significant hyponatremia | 0 (0.0) | 2 (0.6) | 0 (0.0) | 1 (0.4) |
| Prolonged use (>1 week) of first-generation antihistamines | 1 (0.6) | 2 (0.6) | 1 (0.8) | 5 (1.8) |
| Gastrointestinal System | | | | |
| PPI at full therapeutic dose >8 weeks | 13 (7.3) | 35 (10.5) | 11 (9.0) | 37 (13.2) |
| Anticholinergic antispasmodic drugs with chronic constipation | 0 (0.0) | 0 (0.0) | 0 (0.0) | 1 (0.4) |
| Respiratory System | | | | |
| Systemic instead of inhaled corticosteroids for maintenance of moderate/severe COPD | 4 (2.2) | 6 (1.8) | 0 (0.0) | 2 (0.7) |
| Musculoskeletal System | | | | |
| NSAID with moderate/severe hypertension | 4 (2.2) | 7 (2.1) | 2 (1.6) | 5 (1.8) |
| NSAID with heart failure | 6 (3.4) | 7 (2.1) | 3 (2.5) | 4 (1.4) |
| Long-term NSAID (>3 months) for symptom relief of OA | 4 (2.2) | 11 (3.3) | 1 (0.8) | 8 (2.8) |
| Warfarin and NSAID together | 0 (0.0) | 1 (0.3) | 0 (0.0) | 1 (0.4) |
| NSAID with chronic renal failure | 0 (0.0) | 0 (0.0) | 1 (0.8) | 0 (0.0) |
| Long-term corticosteroids (>3 months) as monotherapy for RA or OA | 1 (0.6) | 9 (2.7) | 2 (1.6) | 7 (2.5) |
| Long-term NSAID or colchicine for chronic treatment of gout without allopurinol C-I | 0 (0.0) | 0 (0.0) | 1 (0.8) | 0 (0.0) |
| Urogenital System | | | | |
| Bladder anticholinergic medications with dementia | 1 (0.6) | 0 (0.0) | 1 (0.8) | 0 (0.0) |
| Anticholinergic medications with glaucoma | 2 (1.1) | 3 (0.9) | 2 (1.6) | 7 (2.5) |
| Anticholinergic medications with constipation | 7 (3.9) | 6 (1.8) | 8 (6.6) | 12 (4.3) |
| Anticholinergic medications with prostatism | 0 (0.0) | 2 (0.6) | 1 (0.8) | 1 (0.4) |
| β-blockers in those with DM and frequent hypoglycemic episodes i.e. ≥1 per month | 1 (0.6) | 0 (0.0) | 0 (0.0) | 0 (0.0) |
| Fall-inducing Drugs | | | | |
| Benzodiazepines | 4 (2.2) | 20 (6.0) | 4 (3.3) | 18 (6.4) |
| Neuroleptic medications | 2 (1.1) | 8 (2.4) | 0 (0.0) | 7 (2.5) |
| First-generation antihistamines | 1 (0.6) | 3 (0.9) | 1 (0.8) | 5 (1.8) |
| Vasodilator drugs with persistent postural hypotension (>20mmHg drop in SPB) | 0 (0.0) | 0 (0.0) | 0 (0.0) | 0 (0.0) |
| Long-term opiates in those with recurrent falls | 10 (5.6) | 19 (5.7) | 8 (6.6) | 18 (6.4) |
| Opiates | | | | |
| Regular opiates for >2 weeks in those with chronic constipation without laxatives | 6 (3.4) | 8 (2.4) | 0 (0.0) | 0 (0.0) |
| Duplicate Prescribing | | | | |
| Any duplicate drug class prescription | 8 (4.5) | 7 (2.1) | 2 (1.6) | 6 (2.1) |
| Total PIMs | 119 | 223 | 83 | 209 |

Key: CCB, Calcium channel blocker; C-I, contraindication; COPD, chronic obstructive pulmonary disease; DM, diabetes mellitus; hx, history; MS, musculoskeletal system; NA, not applicable; NSAID, Non-steroidal anti-inflammatory drug; NYHA, New York Heart Association; OA, osteoarthritis; RA, rheumatoid arthritis; PIMs, potentially inappropriate medicines; PPI, proton pump inhibitor; SPB, systolic blood pressure; SSRl, Selective serotonin re-uptake inhibitor; TCA, Tricyclic antidepressant.

**Table S4 Potential prescribing omissions (PPOs) identified by START, for all individuals enrolled in LiLACS NZ at 12-months and 24-months**

| START Criteria | 12-months  (n=510) | | 24-months  (n=403) | |
| --- | --- | --- | --- | --- |
|  | Māori  (n=178) | Non-Māori  (n=332) | Māori  (n=122) | Non-Māori  (n=281) |
| Cardiovascular System |  | | | |
| Warfarin or aspirin in atrial fibrillation | 11 (6.2) | 17 (5.1) | 6 (4.9) | 26 (9.3) |
| Aspirin or clopidogrel with a documented hx of atherosclerotic coronary/cerebral/peripheral vascular disease in sinus rhythm | 1 (0.6) | 5 (1.5) | 1 (0.8) | 1 (0.4) |
| Antihypertensive therapy where SPB consistently >160 mmHg | 9 (5.1) | 24 (7.2) | 2 (1.6) | 16 (5.7) |
| Statin therapy with a documented hx of coronary/cerebral/peripheral vascular disease, where the patient's functional status remains independent for activities of daily living and life expectancy is greater than 5 years | 12 (6.7) | 45 (13.6) | 12 (9.8) | 21 (7.5) |
| ACE inhibitor with chronic heart failure | 31 (17.4) | 28 (8.4) | 18 (14.8) | 29 (10.3) |
| ACE inhibitor with acute myocardial infarction | 27 (15.2) | 34 (10.2) | 16 (13.1) | 31 (11.0) |
| β-Blocker with chronic stable angina | 7 (3.9) | 12 (3.6) | 2 (1.6) | 4 (1.4) |
| Central Nervous System |  | | | |
| Antidepressant drug in presence of moderate/severe depressive symptoms lasting ≥3 months | 24 (13.5) | 27 (8.1) | 13 (10.7) | 19 (6.8) |
| Gastrointestinal System |  | | | |
| Fibre supplement for chronic, symptomatic diverticular disease with constipation | 5 (2.8) | 3 (0.9) | 5 (4.1) | 9 (3.2) |
| Musculoskeletal System |  | | | |
| Bisphosphonates in patients taking maintenance corticosteroid therapy | 23 (12.9) | 34 (10.2) | 18 (14.8) | 46 (16.4) |
| Calcium and vitamin D supplement in patients with known osteoporosis | 33 (18.5) | 68 (20.5) | 22 (18.0) | 57 (20.3) |
| Endocrine System |  | | | |
| Metformin with type 2 DM ± metabolic syndrome (in the absence of renal impairment) | 19 (10.7) | 28 (8.4) | 12 (9.8) | 25 (8.9) |
| ACE inhibitor or angiotensin receptor blocker in diabetes with nephropathy, i.e. overt urinalysis proteinuria or microalbuminuria (>30 mg/24 hours) ±serum biochemical renal impairment | 12 (6.7) | 18 (5.4) | 9 (7.4) | 15 (5.3) |
| Antiplatelet therapy in DM with coexisting major cardiovascular risk factors | 1 (0.6) | 6 (1.8) | 2 (1.6) | 7 (2.5) |
| Statin therapy in DM if coexisting major cardiovascular risk factors present | 2 (1.1) | 7 (2.1) | 2 (1.6) | 4 (1.4) |
| Total PPOs | 217 | 356 | 140 | 310 |

Key: ACE, angiotensin converting enzyme; DM, diabetes mellitus; hx, history; NA, not applicable; PPOs, potential prescribing omissions; SPB, systolic blood pressure.

**Table S5 The prevalence of Potentially inappropriate medicines (PIMs) within each physiological system, identified by STOPP, for all individuals enrolled in LiLACS NZ at 12-months and 24-months**

|  | 12-months  (n=510) | | | 24-months  (n=403) | | |
| --- | --- | --- | --- | --- | --- | --- |
|  | **Māori**  **(n=178)** | **Non-Māori**  **(n=332)** | **P value** | **Māori**  **(n=122)** | **Non-Māori**  **(n=281)** | **P value** |
| Cardiovascular System  n (%) | 28 (15.7) | 40 (12.0) | 0.24 | 20 (16.4) | 31 (10.9) | 0.14 |
| Central Nervous System  n (%) | **7 (3.9)** | **29 (8.7)** | **0.04** | 10 (8.2) | 32 (11.3) | 0.36 |
| Gastrointestinal System  n (%) | 13 (7.3) | 35 (10.5) | 0.23 | 11 (9.0) | 38 (13.4) | 0.20 |
| Respiratory System  n (%) | 4 (2.2) | 6 (1.8) | 0.76 | 0 (0.0) | 2 (0.7) | 1.00 |
| Musculoskeletal System  n (%) | 12 (6.7) | 26 (7.8) | 0.66 | 8 (6.6) | 16 (5.6) | 0.74 |
| Urogenital System  n (%) | 10 (5.6) | 10 (3.0) | 0.15 | 10 (8.2) | 19 (6.7) | 0.61 |
| Endocrine System  n (%) | 1 (0.6) | 0 (0.0) | 0.35 | 0 (0.0) | 0 (0.0) | NA |
| Fall-inducing Drugs  n (%) | 15 (8.4) | 41 (12.3) | 0.17 | 11 (9.0) | 38 (13.4) | 0.20 |
| Opiates  n (%) | 6 (3.4) | 8 (2.4) | 0.57 | 0 (0.0) | 0 (0.0) | NA |
| Duplicate Prescribing  n (%) | 8 (4.5) | 7 (2.1) | 0.13 | 2 (1.6) | 6 (2.1) | 1.00 |

**Table S6 The** **prevalence of Potential prescribing omissions (PPOs) within each physiological system, identified by START, for all individuals enrolled in LiLACS NZ at 12-months and 24-months**

|  | 12-months  (n=510) | | | 24-months  (n=403) | | |
| --- | --- | --- | --- | --- | --- | --- |
|  | **Māori**  **(n=178)** | **Non-Māori**  **(n=332)** | **P value** | **Māori**  **(n=122)** | **Non-Māori**  **(n=281)** | **P value** |
| Cardiovascular System  n (%) | **162 (91.0)** | **189 (56.9)** | **<0.01** | 44 (36.1) | 94 (33.5) | 0.61 |
| Central Nervous System  n (%) | 73 (41.0) | 117 (35.2) | 0.20 | 13 (10.7) | 19 (6.8) | 0.18 |
| Gastrointestinal System  n (%) | 5 (2.8) | 3 (0.9) | 0.14 | 5 (4.1) | 9 (3.2) | 0.77 |
| Musculoskeletal System  n (%) | 52 (29.2) | 95 (28.6) | 0.89 | 37 (30.3) | 95 (33.8) | 0.49 |
| Endocrine System  n (%) | 26 (14.6) | 37 (11.1) | 0.26 | 16 (13.1) | 30 (10.7) | 0.48 |
